# Microbioma and probiotics: from gut to Mars^☆^

**DOI:** 10.1016/j.bjorl.2017.10.004

**Published:** 2017-10-27

**Authors:** Bruno Acatauassú Paes Barreto

**Affiliations:** a Universidade do Estado do Pará (UEPA), Belém, PA, Brazil; b Centro Universitário do Estado do Pará (CESUPA), Belém, PA, Brazil

Just as human beings, who have been trying to discover a biological substrate in the stars and other planets for decades in order to find a viable alternative for survival, it is possible that many microorganisms, especially bacteria and some viruses, have actually done this successfully for millennia. The result is that today they maintain a mutual relationship of coexistence with human beings. Thus, we have microorganisms inhabiting the most diverse sites of the human body, where the intestinal microbioma plays a fundamental role for the physiological balance and survival of our species.

What level of “aggression” is required for these microorganisms to take over their new hosts? What is the best way to do this? And how do they, even being “strange creatures”, gain the benefit of immune tolerance? These are questions that have long been shrouded in a cloud of uncertainty, and which probably passed through the minds of Nissle and Metchnikoff, but which have been partly clarified in recent decades.

Studies on germ-free animals have confirmed the existence of the neuro-immuno-endocrine axis, and the importance, or rather, the necessity of having a proper and early colonization of the microbiota for the morpho-physiological development of these respective organic systems. But how early does this colonization need to occur? Until a few years ago, according to classical studies and the scientific consensus, at birth every human being had a totally sterile intestinal tract and contents, and the bacterial contamination only occurred a few hours after childbirth. However, with the development of new techniques for identifying these microorganisms, many studies have detected the presence of bacterial DNA, such as some species of *lactobacilli*, *bifidobacteria*, *enterococci* and *clostridia*, in the womb, placenta and amniotic fluid of pregnant women, as well as in the meconium of newborns. These findings, which still need to be reproduced, offer a possibility for the use of probiotic strains in pre and perinatal prevention strategies for maladies such as allergies and metabolic or behavioral disorders.^1^, ^2^

The rationale for these new perspectives of intestinal microbiota modulation by probiotic strains, could be to combat dysbiotic processes that reach children early, especially in the first stages of colonization and maturation of the intestinal microbiota, either by pre or perinatal factors. These dysbiotic processes could include the use of antibiotics during pregnancy, childbirth or the puerperium, and the higher prevalence of cesarean deliveries; postnatal factors, such as shorter duration of breastfeeding and the consumption of a qualitatively inadequate diet, as well as the indiscriminate use of antibiotics in the pediatric age group are also factors to be considered. Dysbiosis could be related to the exponential increase of non-communicable diseases in childhood, both in the short term during infancy, or the long term during adolescence or adulthood.^2^

By definition probiotics are “*live microorganisms that when administered in adequate amounts confer a health benefit on the host.*” However, this is a broad definition that does not specify the strains, routes of administration, targets and possible effects on human health. Therefore, understanding of the specificity of strains is essential in this context, and the scientifically proven effects achieved by one bacterial strain, cannot be attributed to other strains, or even combinations of them, as well as to other genera of microorganisms.

Historically, from the beginning of the 20th century, when Ellie Metchnikoff attributed the longevity of Bulgarian peasants to a rich fermented milk diet containing generous amounts of *Lactobacillus bulgaricus*, a number of other species of lactobacilli have been shown to possess probiotic effects including: *L. acidophilus*, *L. rhamnosus*, *L. reuterii*, *L. casei*, *L. fermentum*, *L. gasseri*, *L. johnsonii*, *L. paracasei* and *L. plantarum*. Similarly there are other probiotic active acidlactic bacteria (bifidobacteria) such as *B. adolescentis*, *B. animalis*, *B. bifidum*, *B. breve* and *B. longum*. Other microorganisms, such as some non-acidlactic bacilli (*Bacillus clausii* and *Bacillus coagulans*), non-pathogenic species of *Escherichia coli* and some fungi such as *Saccharomyces boulardii* also exhibit probiotic activity. More recently, new mutant elements of the intestinal microbiota have shown probiotic capacity and have been considered a promising new generation, among them *Akkermansia muciniphila*, *Faecalibacterium prausnitzii*, *Roseburia* spp. and *Eubacterium hallii*.^3^

Because of this, the increase of publications on probiotics in most medical specialties has been exponential, both in basic research and in clinical studies. This has generated several subsequent analyses, utilizing guides, simple reviews, systematic reviews of the literature or meta-analyses. Recently he World Allergy Organization (WAO) suggested the following recommended uses for probiotics: first, in pregnant and lactating women at risk of having an atopic child, and second, in infants with the same risk profile for development of allergies. The World Allergy Organization (WAO) would consider the use of probiotics as a prevention strategy for the appearance of atopic dermatitis and consequent development of atopic march, with subsequent onset of allergic rhinitis and/or asthma in children at risk. Such recommendations triggered academic criticism because of lack of strain specificity in these indications. However, as a pediatrician and clinical immunologist, I consider these recommendations to be correct and courageous, since they represent a position based on numerous studies with a high level of scientific evidence. So why not indicate specific strains? The answer is simple: because we do not yet have enough studies with the same design, same type of strain and similar endpoints. For example, in the otorhinolaryngological setting, a recent Cochrane systematic review involving 3720 participants (adults and children) showed that probiotics are statistically able to reduce upper respiratory tract infections (URT's), duration, antibiotic use and school/work absenteeism. However, the authors themselves acknowledge the fragility of these findings when analyzing studies with different types of strains, in different populations. That is, in this case “the means would not justify the endpoints.”^4^, ^5^

So, in conclusion, I believe that the search for the unknown is part of human nature, and while, at the spatial level, Mars brings us promising information, but still far from serving as the next human colony. On the other hand, as the intestinal microbiota is increasingly mapped, with identification of new species, new interfaces and mechanisms it will confirm its importance, and that of probiotics, as strategies for prevention and treatment of many diseases that affect our modern society.

## Conflicts of interest

The author declares no conflicts of interest.

## References

[1] Perez-Muñoz M.E., Arrieta M.C., Ramer-Tait A.E., Walteet J. (2017). A critical assessment of the “sterile womb” and “in utero colonization” hypotheses: implications for research on the pioneer infant microbiome. Microbiome.

[2] Nuriel-Ohayon M., Neuman H., Koren O. (2016). Microbial changes during pregnancy, birth, and infancy. Front Microbiol.

[3] Guarner F., Khan A.G., Garish J., Eliakim R., Gangl A., Thomson A. (2011). Diretrizes Mundiais da Organização Mundial de Gastroenterologia. Probióticos e prebióticos.

[4] Fiocchi A., Pawankar R., Cuello-Garcia C., Ahn K., Al-Hammadi S., Agarwal A. (2015). World allergy organization-McMaster University Guidelines for Allergic Disease Prevention (GLAD-P): probiotics. World Allergy Organ J.

[5] Hao Q., Dong B.R., Wu T. (2015). Probiotics for preventing acute upper respiratory tract infections. Cochrane Database Syst Rev.

